# Young and early‐onset dilated cardiomyopathy with malignant ventricular arrhythmia and sudden cardiac death induced by the heterozygous LDB3, MYH6, and SYNE1 missense mutations

**DOI:** 10.1111/anec.12840

**Published:** 2021-05-05

**Authors:** Ting Zhao, Yuting Ma, Zuoquan Zhang, Jianzhong Xian, Xiaojing Geng, Feng Wang, Jiana Huang, Zhe Yang, Yi Luo, Yubi Lin

**Affiliations:** ^1^ The First Hospital Affiliated to Jinan University The First People's Hospital of Guangzhou Guangzhou China; ^2^ Department of Cardiology The Cardiovascular Center Interventional Medical Center Guangdong Provincial Key Laboratory of Biomedical Imaging and Guangdong Provincial Engineering Research Center of Molecular Imaging The Fifth Affiliated Hospital Sun Yat‐sen University Zhuhai China; ^3^ BGI Education Center University of Chinese Academy of Sciences Shenzhen China; ^4^ Guangdong Academy of Medical Sciences Guangdong Geriatrics Institute Guangdong Cardiovascular Institute Guangdong Provincial People's Hospital Guangzhou China; ^5^ Reproductive Center The Six Affiliated Hospital Sun Yat‐sen University Guangzhou China

**Keywords:** arrhythmia, cardiomyopathy, gene, sudden cardiac death

## Abstract

**Background:**

The whole exome sequencing (WES) with targeted gene analysis is an effective diagnostic tool for cardiomyopathy. The early‐onset sudden cardiac death (SCD) was commonly associated with dilated cardiomyopathy (DCM) induced by pathogenic genetic mutations.

**Methods:**

In a Chinese Han family, the patient of 24 years old occurred with early‐onset and DCM and died of SCD associated with ICD storms induced by repetitive ventricular tachycardia/fibrillation (VT/F). Genomic DNA samples of peripheral blood were conducted for WES and Sanger sequence. Then, we performed bioinformatics analysis for 200 genes susceptible to cardiomyopathies and arrhythmias. Further, we analyzed how the potential pathogenic mutations affecting the secondary structure, hydrophobicity, and phosphorylation of amino acids, protein properties, and their joint pathogenicity by ProtParam, SOPMA, and ORVAL algorisms. The protein–protein interaction was analyzed by STRING algorism.

**Results:**

The mutations of LDB3 p.M456R, MYH6 p.S180Y, and SYNE1 p.S4607F were identified as “Damaging/Deleterious.” The SYNE1 (p.S4607F) increased one of alpha helix and decreased one of beta sheet. The LDB3 (p.M456R) reduced one of beta sheet and increased one of beta turn. The MYH6 (p.S180Y) decreased two of beta sheets and four of beta turns, but significantly increased twelve coils. The hydrophobicity of amino acid residues and their adjacent sequences were decreased by LDB3 (p.M456R) and MYH6 (p.S180Y), and significantly increased by SYNE1 (p.S4607F). The mutations of LDB3 (p.M456R), SYNE1 (p.S4607F), and MYH6 (p.S180Y) resulted in the phosphorylation changes of the corresponding amino acid sites or the nearby amino acid sites. The pairwise combinations of LDB3, MYH6, and SYNE1 mutations have the high probability of causing disease, especially the highest probability for SYNE1 and LDB3 mutations. There was obviously indirect interaction of the proteins encoded by SYNE1, LDB3, and MYH6.

**Conclusions:**

The multiple heterozygous mutations of SYNE1, LDB3, and MYH6 may be associated with young and early‐onset of DCM and SCD.

**What is new?**

- We firstly reported that the multiple heterozygous mutations of SYNE1 (p.S4607F), LDB3 (p.M456R), and MYH6 (p.S180Y) may be associated with young and early‐onset dilated cardiomyopathy, malignant ventricular arrhythmia, and sudden cardiac death.
- The mutations of LDB3, MYH6, and SYNE1 were identified as “Damaging/Deleterious,” and abnormally changed the secondary structure, hydrophobicity, and phosphorylation of amino acids, which therefore increased the joint pathogenicity.
- There was obviously indirect interaction of the proteins encoded by SYNE1, LDB3, and MYH6.

## 1 BACKGROUND

Cardiomyopathies are classified as dilated cardiomyopathy (DCM), hypertrophic cardiomyopathy (HCM), restrictive cardiomyopathy (RCM), and arrhythmogenic right ventricular cardiomyopathy/dysplasia (ARVC/D). DCM is a rare cardiac disease characterized by left ventricular enlargement, reduced left ventricular contractility, and impaired systolic function, which can lead to sudden cardiac death (SCD), with a reported frequency of 46% (Cecchi et al., 2012; Fu & Eisen, 2018). Hereditary DCM is present in almost 50% of reported cases, mostly inherited in an autosomal dominant pattern, although some cases reported with X‐linked, autosomal recessive, and mitochondrial DNA inheritance (McNally et al., 2013). The hereditary DCM is complicated by incomplete penetrance, high variability in age of onset and disease progression, and high genetic heterogeneity (Haas et al., 2015). Cardiomyopathies are associated with >100 known genes, including genes encoding for sodium and potassium channel in sarcolemma, dystrophin proteins in cytoplasm, myofibril with sarcomere/Z‐disk, thin/thick filament, RNA‐binding protein, cytoskeletal proteins, desmosome mitochondria, sarcoplasmic reticulum, and nucleus envelope (McNally et al., 2013; Reichart et al., 2019). Following with the advantage of targeted sequencing technology, multiple mutations are increasingly reported and are often associated with high disease severity. Notably, 12.8% of DCM has at least three known mutations (Haas et al., 2015).

The whole exome sequencing (WES) with targeted gene analysis is an effective diagnostic tool for patients with DCM in a clinical setting. The pathogenic or likely pathogenic mutations were identified in 12% of DCM by WES (Ramchand et al., 2020). The genetic testing was considered valuable for future SCD risk factor stratification and genetic counseling for DCM. For example, desmosome and LAMA gene mutations increased the risk of arrhythmia and SCD for DCM (Gigli et al., 2019; Li et al., 2020). Here, we aimed to identify novel mutations and genes responsible for young and early‐onset DCM with SCD using WES. In doing so, we identified multiple potential pathogenic mutations of LDB3, MYH6, and SYNE1 genes in this patient.

## 2 METHODS

### 2.1 Ethical compliance

This study was approved by the Guangdong Medical Institutional Review Board and Medical Ethics Committees [No. GDREC2016001H (R1)]. Detailed clinical information was collected. The clinical information included family history, age of presentation, initial symptoms of ventricular tachycardia, physical examination, electrocardiograms (ECGs), and echocardiograms based on their informed consents.

### 2.2 Whole exome sequencing

Genomic DNA samples of patients were isolated from peripheral blood using a standard DNA extraction protocol. The isolated genomic DNA was then fragmented into 150–200 bp and subjected to DNA library preparation using established Illumina paired‐end protocols. Adaptor‐ligated libraries were amplified via PCR. A portion of each library was used to create an equimolar pool. Each pool was amplified to enrich targets sequenced by the Agilent SureSelectXT Target Enrichment System (Agilent Technologies Inc., Santa Clara, CA, USA). Whole exome capture was performed with the Agilent SureSelectXT Human All Exon 50 Mb Kit (Agilent Technologies Inc.) following the manufacturer's protocol. The exome‐enriched libraries were sequenced with the Illumina Hiseq 2000 platform (Illumina, San Diego, CA, USA) according to the manufacturer's instructions, and 100 bp paired‐end sequencing reads were generated. Each sample was sequenced per lane to obtain an average theoretical depth of 100×.

### 2.3 Read mapping, variant detection, and functional annotation

Raw reads were collected for quality control, in which low‐quality reads were filtered, and 3′/5′ adapters were trimmed using the Trim Galore program. Clean reads were aligned to the human reference genome (University of California Santa Cruz, UCSC build hg19) using the Burrows‐Wheeler Aligner (BWA) program. The quality scores were recalibrated, and reads were realigned to the reference genome using the Genome Analysis Toolkit (GATK) software package. Following the exclusion of duplicate reads, insertion‐deletions (InDels) and single‐nucleotide polymorphisms (SNPs) were called using the GATK or Sequence Alignment/Map tools (SAM tools).

SNPs and InDels were annotated using a pipeline in which all insertion, and deletion mutations were occurring in coding regions. InDels were considered damaging, and nonsynonymous SNPs predicted by SIFT (http://sift.jcvi.org/www/) (Kumar et al., 2009) and PolyPhen‐2 (Polymorphism Phenotyping v2, http://genetics.bwh.harvard.edu/pph2/) (Adzhubei et al., 2010). The mutations were screened in approximately 200 genes (shown in our previous study (Lin et al., 2017)), associated with hereditary arrhythmias and cardiomyopathies. The filtering criteria for mutation inclusion were as follows: same mutations in the WES data; missense, nonsense, and InDel mutations; and SNPs with a minor allele frequency not more than 0.01, according to the NCBI SNP database (Smigielski et al., 2000; Via et al., 2010). We subsequently focused the genetic investigation on rare functional mutations, defined as previously unreported or with a very low of minor allele frequency (MAF < 0.1%) in the European population of the ExAC database. Meanwhile, these mutations predicted to alter the protein sequence (Di Resta et al., 2015).

### 2.4 Sanger sequencing

When the suspected pathogenic mutations were obtained in each step, they were screened again using Sanger sequencing in the other members of the family. The following primers designed with Primer Premier 5.0 were used and showed as follows (forward primer, reverse primer, length, annealing temperature).

MYH6: AGGGTGTAGTTGGGAGGAGAG, GAGGGGAGGGTTAGGGGTA, 406 bp, 57.0°C.
LDB3: CTTCCTGATTTTCTTTATTGT, GAGTTGCTTTCTCTGTTACC, 497 bp, 48.5℃.
SYNE1: AAGAGTGAAGTGCTGGGGAAG, AGGGGTCAAGGTGTGGGG, 961 bp, 58.5°C.
COL3A1: GCATTCCTTCGACTTCTC, TATTTCAACTGGTTTTCATCT, 404 bp, 49.5°C.
PSEN2: AGGTCCTTGTGCTCCTTTTTC, TCATCATTACTTCCCTTCTCCC, 301 bp, 57.5°C.
OBSCN: CATCCTCCAGTCTCCTTTCCC, CCCCACCTTACCTGCCCTT, 608 bp, 61.5°C.
PKP2: CAAGTCTCCAGGTGTCCGC, TTTCCACAAGCAAGTCGGTC, 671 bp, 58.4°C.
TTN, CAACAGGTCTTCGTCGGATT, CCTCTTGCTTGGGTATTTTCAT, 877 bp, 57.5°C.
TTN: TAAAATGGGAGAAAGATGGTCA,TTGGGATGTGATAGGTTGAATA, 1,023 bp, 56.5°C.
COL1A2: AGCCCCTCCCACTAAAGAC,AAAATACACCACACGATACAACT, 909 bp, 55.0°C.

A 50 μl polymerase chain reaction (PCR) mix was made up of 10× PCR buffer (100 mM Tris‐HCl, pH 8.3, 500 mM KCl), 125 μM dNTPs, 10 pmol of each primer, 2 U of Taq DNA polymerase, and 100 ng genomic DNA templates. The following steps were used for the PCR: 94°C for 3 min and 35 cycles (of 94°C for 30 s, 55°C for 1 min, and 72°C for 1 min), followed by 72°C for 10 min. The PCR products were purified using a Wizard Miniprep Kit. The purified PCR products were sequenced with an ABI PRISM BigDye Terminator Cycle Sequencing Ready Reaction Kit using standard conditions. The sequencing products were purified using ethanol/EDTA/sodium acetate precipitation and analyzed on an ABI 3730xl Automated DNA Sequencer.

### 2.5 Myocardial biopsy

The right internal jugular vein was punctured, and a 7‐Fr vascular sheath was inserted. A long, flexible catheter with forceps in the head end was inserted into a vein and threaded up into the heart while monitored by X‐ray. Five small snips of muscle at the apical septum of the right ventricle were grabbed. Some cardiac tissues, which were fixed with formalin solutions, then dehydrated, paraffin‐embedded and sliced, were used for HE and Masson staining. Some cardiac tissues were fixed with glutaraldehyde and osmic acid, dehydrated, resin embedded and sliced, and then prepared for uranyl acetate and lead nitrate staining and analyzed using a transmission electron microscope.

### 2.6 Physical‐chemical analysis and secondary structure prediction

ProtParam is a tool which allows the computation of various physical and chemical parameters for a given protein for a user entered protein sequence. The computed parameters include the molecular weight, theoretical pI, amino acid composition, atomic composition, extinction coefficient, estimated half‐life, instability index, aliphatic index, and grand average of hydropathicity (GRAVY) (Wilkins et al., 1999). The secondary structures of wild‐type and potential pathogenic mutations were predicted by SOPMA algorism (Geourjon & Deléage, 1995).

### 2.7 Joint pathogenicity and protein–protein interaction analysis

ORVAL (https://orval.ibsquare.be) is the first web bioinformatics platform for the exploration of predicted candidate disease‐causing variant combinations, aiming to aid in uncovering the causes of oligogenic diseases (i.e., diseases caused by variants in a small number of genes). This tool integrates innovative machine learning methods for combinatorial variant pathogenicity prediction, further external annotations and interactive and exploratory visualization techniques (Renaux et al., 2019). The STRING online tool (https://string‐db.org/) was used to extract the target protein information from PPI database to explore any direct and indirect protein–protein interactions (PPIs) in the target genes (Szklarczyk et al., 2019).

## 3 RESULTS

### 3.1 Familial characteristics

**FIGURE 1:**
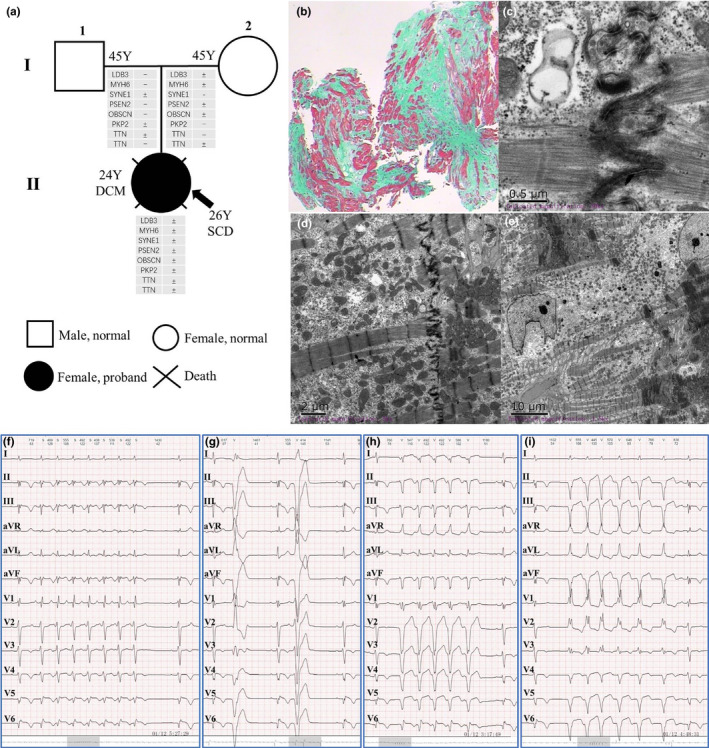
The pedigree, myocardial biopsy, and electrocardiogram. (a) The potential pathogenic mutations susceptible to cardiomyopathy and arrhythmia among the family members. (b) Myocardial HE staining. (c‐e) Myocardial electron‐microscopic examination. (f‐i) Electrocardiograms

**FIGURE 2:**
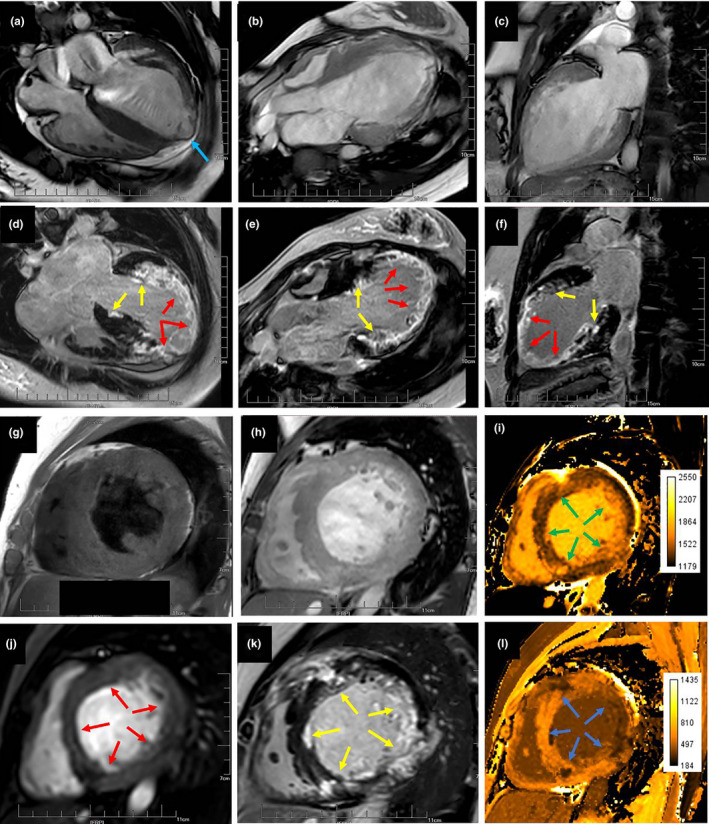
Cardiac magnetic resonance imaging. The first line (a‐c) was the film sequence (balance TFE). The second line (d‐f) was delay enhanced sequence (DCE PSIR). Figure (a‐f) showed that the anterior wall, lateral wall, inferior wall, and septum of left ventricle were extensively involved. Some districts were transmurally involved (red arrow). Part of districts was mainly involved in endocardium (yellow arrow). The middle and apical segment were most obviously involved. The apical myocardium was obviously thinned and ventricular aneurysm was formed (blue arrow). Figure (g), T1W TSE. Figure (h), Balance TFE. Figure (i), MOLLI T1 native. Figure (j), Dynamic sTFE. Figure (k), PSIR TFE. Figure (l), MOLLI T1 DCE. The short‐axis view of middle segment showed thickening of interventricular septum (19 mm), scattered and multiple lesions in the anterior wall, lateral inferior wall and inferior interventricular septum of left ventricle. On plain scan, T1W showed low signal, while T1 value was significantly prolonged (green arrow). The first pass perfusion showed low perfusion (red arrow). The delayed enhancement showed the high enhancement (yellow arrow). The delayed enhancement T1 value was significantly shortened (blue arrow)

In a Chinese Han family, a 24‐year‐old female patient repeatedly (II: 1, proband) presented with palpitations, dizziness, fatigue, sweating, and then syncope after exertion for 1 year. She woke up on her own for about 1 min (Figure 1a). During this period, ventricular tachycardia occurred and was converted into sinus rhythm by intravenous application of amiodarone. She was hospitalized in January 2016. The dynamic electrocardiogram (Figure 1f‐i) illustrated that: rSr' type of QRS waves in II, III and aVF leads, and obvious notch in S waves; incomplete right bundle branch block, poor increases of R waves in precordial lead, rS and Qr type of QRS waves in V_4_‐V_6_ leads; T wave inversion in II, III, aVF, and V_3_‐V_6_ leads; short episodes of atrial tachycardia (Figure 1f); multi‐source premature ventricular contraction (Figure 1g); ventricular tachycardia originated from the apical septum of right and left inferior wall (Figure 1h,i). Echocardiography showed that the ventricular apical aneurysm, mild mitral and tricuspid regurgitation, and decreased left ventricular systolic function. Cardiac magnetic resonance imaging (Figure 2) indicated that bilateral ventricular myocardial thickening and scattered fibrosis, especially the myocardium in middle and apical free wall of left ventricle was the most serious, showing extensive fibrosis and local thinning; severe left ventricular enlargement and severe reduction of global systolic function; mild enlargement of right ventricle and mild reduction of global systolic function; normal size of right atrium and slight enlargement of left atrium; mild regurgitations were seen in tricuspid, mitral, and pulmonary valves. In January 2016, she was implanted with implantable cardioverter‐defibrillator (ICD) and suffered from palpitation and ICD discharge twice, followed by vomiting, dizziness, shortness of breath, fatigue, and numbness at the end of limbs in July 2016. Although therapy with beta blocker and amiodarone, she still occurred with SCD in October 2017, due to frequent ICD storms induced by repetitive ventricular tachycardia/fibrillation (VT/F). Her father (I: 1) and mother (I: 2) had normal echocardiography and electrocardiogram with no clinical symptoms, indicating no history and clinical evidence of cardiomyopathy and arrhythmia.

### 3.2 Pathological features of myocardium

The pathological examination showed that some muscle fibers of myocardial tissue were atrophied and reduced in volume, some muscle fibers were hypertrophic; the nucleus of myocardium was large and deeply stained, and obvious interstitial fiber hyperplasia was found (Figure 1b). The intercalated disk of cardiomyocytes was not connected (Figure 1c‐e), and the local space was widened. The sizes of mitochondria were different, and part of mitochondria was slightly focal vacuolar degeneration. The local myocardial fibrils were dissolved and disappeared, and many glycogen particles were accumulated and filled.

### 3.3 Genetic screening

**FIGURE 3:**
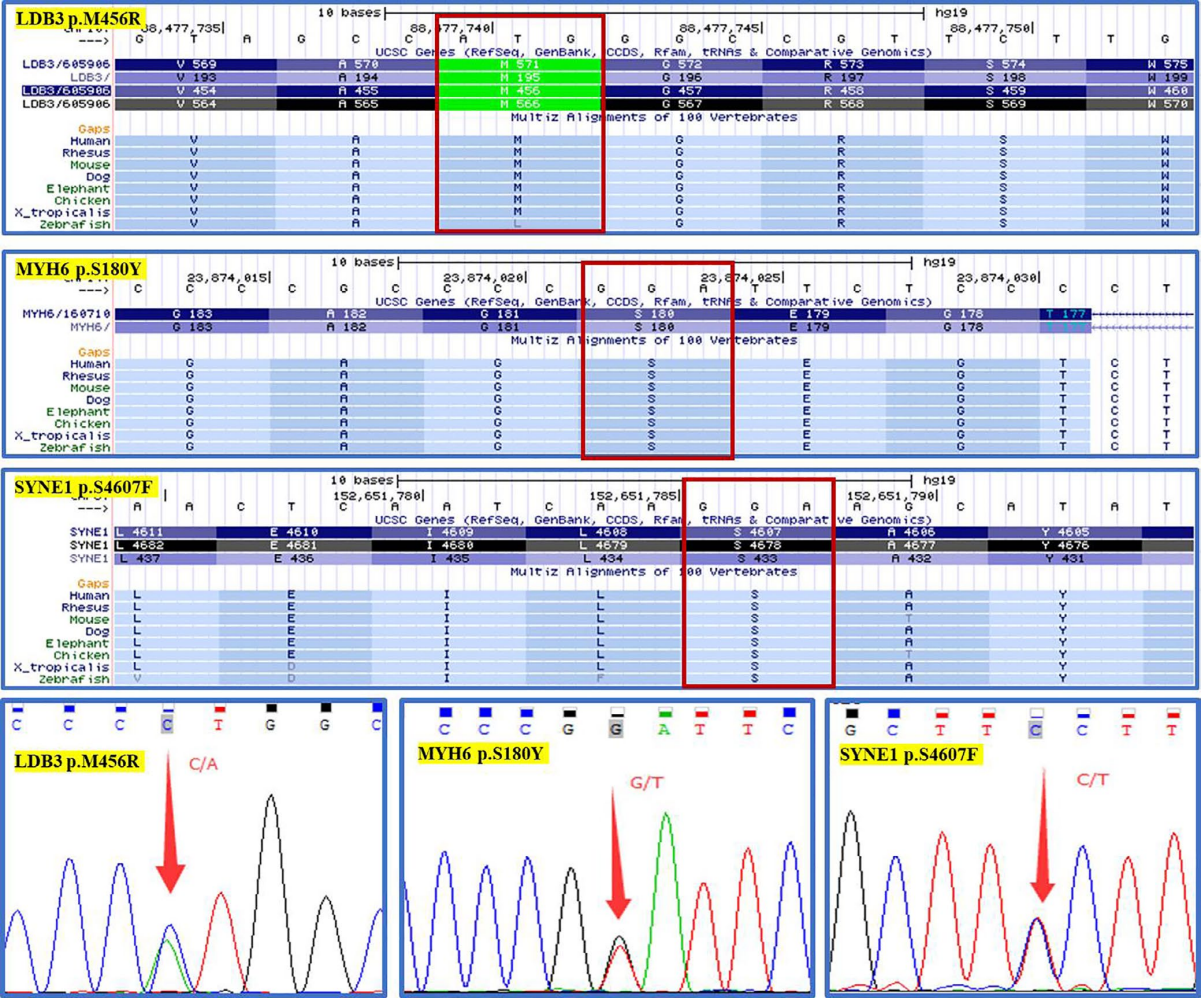
The conservative analysis of the risk genetic mutations

In this family (Figure 1a), a set of candidate genes associated with cardiomyopathies and arrhythmias were screened using the WES data of II: 1. The results (Table 1) showed that II: 1 carried the heterozygous mutations of PSEN2 p.G34S (rs200636353), OBSCN p.N393D, LDB3 p.M456R (rs566463138), PKP2 p.K52R (rs549598534), MYH6 p.S180Y, TTN p.I25488T, TTN p.Y3760H (rs377740664), SYNE1 p.S4607F, COL3A1 p.Q1381H, COL3A1 p.K1391R (rs373015577), and COL1A2 p.F1341I. The mutations of COL3A1 and COL1A2 were verified to be false positive by Sanger sequencing. The mutations of PSEN2 p.G34S, OBSCN p.N393D, PKP2 p.K52R, TTN p.I25488T, and TTN p.I25613T were predicted as “Tolerated/Benign” by SIFT and Polyphen‐2 algorithms. The mutations of LDB3 p.M456R, MYH6 p.S180Y, and SYNE1 p.S4607F were predicted as “Damaging/Deleterious.” According to the population of 1000 Genomes Project (2015 release version), the minor allele frequency (MAF) of LDB3 p.M456R was less than 0.001, while the mutations of MYH6 p.S180Y and SYNE1 p.S4607F were not found. For II: 1 proband, the SYNE1 p.S4607F mutation was inherited from her father (I: 1), while the mutations of LDB3 p.M456R and MYH6 p.S180Y were inherited from her mother (I: 2). The parents of II: 1 had no clinical evidence of cardiomyopathy based on electrocardiogram and echocardiogram examination. Conservative analysis demonstrated that LDB3 p.M456, MYH6 p.S180, and SYNE1 p.S4607 were very conservative among species (Figure 3). The verifications of Sanger sequencing for LDB3 p.M456R, MYH6 p.S180Y, and SYNE1 p.S4607F were showed in Figures 1a and 3.

**TABLE 1:** The potential pathogenic mutations of II: 1

Chr	Location	Gene	Amino acid change	All	AFR	AMR	EAS	EUR	SAS	SIFT	Polyphen2	Protein domain
chr1	227069708	PSEN2	NM_000447:exon4:c.G100A:p.G34S	0.0011	–	–	0.005	–	0.001	0.44(T)	0.01(B)	–
chr1	228401330	OBSCN	NM_001098623:exon3:c.A1177G:p.N393D	–	–	–	–	–	–	1.00(T)	0.10(B)	Immunoglobulin subtype
chr10	88477741	LDB3	NM_001080114:exon10:c.T1367G:p.M456R	0.0002	–	–	0.001	–	–	0.00(D)	0.97(D)	Zinc finger, LIM‐type
chr12	33049511	PKP2	NM_001005242:exon1:c.A155G:p.K52R	0.0002	–	–	0.001	–	–	0.17(T)	0.12(B)	–
chr14	23874023	MYH6	MYH6:NM_002471:exon7:c.C539A:p.S180Y	–	–	–	–	–	–	0.00(D)	0.97(D)	Myosin head, motor domain
chr2	179397684	TTN	NM_003319:exon186:c.T76463C:p.I25488T	–	–	–	–	–	–	0.06(T)	0.00(B)	Fibronectin type III
chr2	179615849	TTN	NM_133379:exon46:c.T11278C:p.Y3760H	0.0012	–	–	0.006	–	–	0.60(T)	0.00(B)	
chr2	189875505	COL3A1	NM_000090:exon50:c.G4143T:p.Q1381H	–	–	–	–	–	–	0.07(T)	0.00(B)	Fibrillar collagen, C‐terminal
chr2	189875534	COL3A1	NM_000090:exon50:c.A4172G:p.K1391R	–	–	–	–	–	–	0.04(D)	0.00(B)	
chr7	94059625	COL1A2	NM_000089:exon52:c.T4021A:p.F1341I	–	–	–	–	–	–	1.00(T)	0.11(B)	
chr6	152651787	SYNE1	NM_033071:exon77:c.C13820T:p.S4607F	–	–	–	–	–	–	0.00(D)	0.99(D)	–

Note

The minor allele frequency (MAF) in all population (ALL) or the species of African (AFR), American (AMR), East Asian (EAS), European (EUR), and South Asian (SAS) from 1000genomes (2015 version).

Abbreviations: –, no report; B, benign; Chr, chromosome; D, damaging; T, tolerated.

### 3.4 The prediction of secondary structure and protein properties

Compared with wild type (WT) of MYH6, the total number of positively charged residues (Arg + Lys) was obviously reduced by MYH6 p.S180Y (362 vs. 316). The total number of positively charged residues (Arg + Lys) increased from 54 of LDB3 (WT) to 55 of LDB3 p.M456R (Table 2). The SYNE1 p.S4607F increased one of alpha helix and decreased one of beta sheet. The LDB3 p.M456R reduced one of beta sheet and increased one of beta turn. MYH6 p.S180Y decreased two of beta sheets and four of beta turns, but significantly increased twelve coils (Table 3).

**TABLE 2:** The effects of mutations on the protein properties

Genotype/protein properties	SYNE1 (WT)	SYNE1 (p.S4607F)	LDB3 (WT)	LDB3 (p.M456R)	MYH6 (WT)	MYH6 (p.S180Y)
Number of amino acids	8,748		617		1,939	
Molecular weight	1,005,126.19	1,005,186.29*	66,671.22	66,696.21*	223,734.71	223,810.81*
Theoretical pI	5.36	5.36	8.29	8.39*	5.58	5.58
NO. of negatively charged residues (Asp + Glu)	1,429	1,429	49	49	361	361
No. of positively charged residues (Arg + Lys)	1,114	1,114	54	55*	362	316*
Carbon (C)	43,928	43,934*	2,951	2,952*	9,749	9,755*
Hydrogen (H)	70,825	70,829*	4,544	4,547*	15,839	15,843*
Nitrogen (N)	12,347	12,347	808	811*	2,777	2,777
Oxygen (O)	13,931	13,930*	898	898	3,104	3,104
Sulfur (S)	321	321	30	29*	66	66
Total number of atoms	141,352	141,361*	9,231	9,237*	31,535	31,545*
Ext. coefficient‐1	933,830	933,830	71,375	71,375	116,965	118,455*
Abs 0.1% (1 g/L)	0.929	0.929	1.071	1.070*	0.523	0.529*
Instability index	51.65	51.65	59.09	58.95	46.98	46.84
Aliphatic index	88.80	88.80	60.81	60.81	81.56	81.56
Grand average of hydropathicity (GRAVY)	−0.62	−0.62	−0.42	−0.43*	−0.81	−0.81

Note

Extinction coefficients are in units of M^−1^ cm^−1^, at 280 nm measured in water. Ext. coefficient‐1 and Abs‐1 [Abs 0.1% (=1 g/L)], assuming all pairs of Cys residues form cystines. Ext. coefficient‐2 and Abs‐2[Abs 0.1% (=1 g/L)], assuming all Cys residues were reduced.

Abbreviation: WT, wild type.

* Showed the changes of the parameters.

**TABLE 3:** The effects of mutations on the secondary structure of protein

Genotype/properties	SYNE1 (WT)	SYNE1 (p.S4607F)	LDB3 (WT)	LDB3 (p.M456R)	MYH6 (WT)	MYH6 (p.S180Y)
αhelix	3,845 (77.16%)	3,846 (77.18%)*	132 (21.39%)	132 (21.39%)	1,479 (76.28%)	1,473 (75.97%)*
ß sheet	269 (5.40%)	268 (5.38%)*	107 (17.34%)	106 (17.18%)*	125 (6.45%)	123 (6.34%)*
ß Turn	230 (4.62%)	230 (4.62%)	51 (8.27%)	52 (8.43%)*	73 (3.76%)	69 (3.56%)*
Coil	639 (12.82%)	639 (12.82%)	327 (53.00%)	327 (53.00%)	262 (13.51%)	274 (14.13%)*

* Showed the changes of the parameters.

### 3.5 Hydrophobicity and phosphorylation

The mutations of LDB3 p.M456R, SYNE1 p.S4607F, and MYH6 p.S180Y located in the non‐transmembrane districts of proteins. The hydrophobicity analysis (Table 4) demonstrated that the hydrophobicity of amino acid residues and their adjacent sequences of LDB3 p.M456R and MYH6 p.S180Y decreased, and the change of hydrophobicity induced by LDB3 p.M456R was much greater than that induced by MYH6 p.S180Y. The hydrophobicity of amino acid residues and their adjacent sequences of SYNE1 p.S4607F significantly increased. The change of hydrophobicity score from high to low was LDB3 > SYNE1 > MYH6.

**TABLE 4:** The changes of Hydrophobicity and phosphorylation

*LDB3* (p.M456R)														
Position		450	451	452	453	454	455	456	457	458	459	460	461	462
AA	W	G	P	F	L	V	A	M	G	R	S	W	H	P
	M	G	P	F	L	V	A	R*	G	R	S	W	H	P
HP	W	1.06	1.64	1.39	0.84	0.84	0.80	0.88	0.21	−0.39	−1.24	−1.83	−1.73	−1.77
	M	1.06	1.64	0.68*	0.13*	0.13*	0.09*	0.17*	−0.50*	−1.10*	−1.96*	−2.54*	−1.73	−1.77
PP	W	–	–	–	–	–	–	–	–	–	No	–	–	–
	M	–	–	–	–	–	–	–	–	–	Yes	–	–	–

MYH6 (p.S180Y)														
Position		174	175	176	177	178	179	180	181	182	183	184	185	186
AA	W	I	L	I	T	G	E	S	G	A	G	K	T	V
	M	I	L	I	T	G	E	Y*	G	A	G	K	T	V
HP	W	0.04	0.04	0.34	0.69	0.98	0.43	−0.42	−1.00	−0.46	−0.80	−0.49	−0.83	−1.29
	M	0.04	0.04	0.29*	0.63*	0.92*	0.38*	−0.48*	−1.06*	−0.51*	−0.86*	−0.54*	−0.83	−1.29
PP	W	–	–	–	–	–	–	Yes	–	–	–	–	–	–
	M	–	–	–	–	–	–	No	–	–	–	–	–	–

*SYNE1* (p.S4607F)														
Position		4,607	4,608	4,609	4,610	4,611	4,612	4,613	4,614	4,615	4,616	4,617	4,618	4,619
AA	W	S	L	I	E	L	T	T	Q	S	L	S	E	L
	M	F*	L	I	E	L	T	T	Q	S	L	S	E	L
HP	W	−0.78	−0.53	0.06	−0.23	−0.31	−0.23	−0.82	0.10	0.46	0.77	0.52	0.23	0.74
	M	−0.78	−0.53	0.06	0.17*	0.09*	0.17*	−0.42*	0.50*	0.86*	1.17*	0.92*	0.63*	0.74
PP	W	Yes	–	–	–	–	–	–	–	–	–	–	–	–
	M	No	–	–	–	–	–	–	–	–	–	–	–	–

Abbreviations: AA, amino acids; HP, hydrophobicity; PP, phosphorylation; W, wild type; M, mutation .

* Showed the changes of the parameters.

The phosphorylation analysis showed that the mutations of LDB3 p.M456R, SYNE1 p.S4607F and MYH6 p.S180Y resulted in the phosphorylation changes of the corresponding amino acid sites. The LDB3 p.M456R led to the transformation of 456^th^ amino acid from the non‐phosphorylation site to phosphorylation site. The SYNE1 p.S4607F and MYH6 p.S180Y led to the disappearance of phosphorylation of the corresponding amino acid sites. The reliability of phosphorylation from high to low was SYNE1 > LDB3 > MYH6.

### 3.6 Joint pathogenicity and protein–protein interaction

**FIGURE 4:**
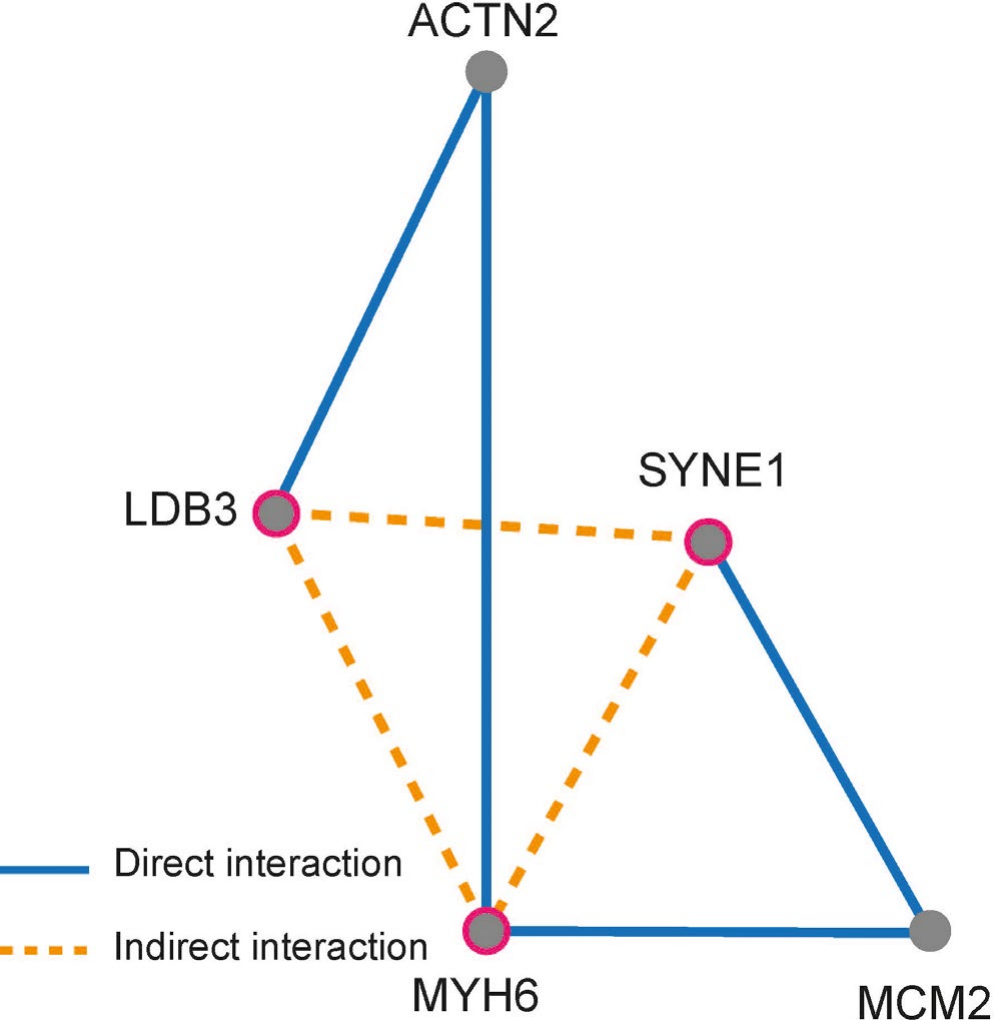
The protein–protein interactions for the risk genes associated with cardiomyopathy. ACTN2, actinin alpha 2; MCM2, minichromosome maintenance complex component 2

We analyzed the joint pathogenicity of candidate pathogenic sites (Table 5), including the genes of LDB3, MYH6, PSEN2, and SYNE1. The pairwise combinations of LDB3 p.M456R, MYH6 p.S180Y, and SYNE1 p.S4607F had the high probability of causing disease, among which the combination of SYNE1 and LDB3 mutations had the highest joint pathogenic probability, and the other two combinations were also very high. The supporting scores of joint pathogenicity of SYNE1/LDB3, SYNE1/MYH6 and LDB3/MYH6 mutations were 99.80, 99.60, and 97.60, respectively. The probability of other genes or other combinations was relatively low. The three proteins corresponding to SYNE1, LDB3, and MYH6 had a real protein–protein interaction relationship (Figure 4), which could form an indirect protein–protein interaction network, while the proteins corresponding to OBSCN and PSEN2 genes had no protein–protein interaction with the proteins corresponding to other genes.

**TABLE 5:** The risk prediction of pairwise‐gene combination

Gene A	Gene B	Gene A alleles	Gene B alleles	CS	SS	PC
LDB3	SYNE1	10:88477741:T:G	6:152651787:G:A	0.83	99.80	D
MYH6	SYNE1	14:23874023:G:T	6:152651787:G:A	0.78	99.60	D
LDB3	MYH6	10:88477741:T:G	14:23874023:G:T	0.68	97.60	D
PSEN2	SYNE1	1:227069708:G:A	6:152651787:G:A	0.57	80.00	D
LDB3	OBSCN	10:88477741:T:G	1:228401330:A:G	0.55	73.20	D
PSEN2	LDB3	1:227069708:G:A	10:88477741:T:G	0.51	60.00	D
SYNE1	OBSCN	6:152651787:G:A	1:228401330:A:G	0.45	36.20	N
MYH6	OBSCN	14:23874023:G:T	1:228401330:A:G	0.35	8.20	N
PSEN2	MYH6	1:227069708:G:A	14:23874023:G:T	0.25	1.00	N
PSEN2	OBSCN	1:227069708:G:A	1:228401330:A:G	0.13	0.00	N

Note

Classification score represented the pathogenicity probability of this combination in Random Forest algorithms. Support score represented the pathogenicity support of this combination in in Random Forest algorithms.

Abbreviations: CS, classification score; SS, support score; PC, predicted class; D, disease‐causing; N, neutral .

## 4 DISCUSSION

### 4.1 LDB3 and cardiomyopathy

LDB3 encodes the ZASP protein in humans and cypher protein in mice. It expresses as a cardiac and skeletal protein in the cytoplasm, which not only stabilizes the Z‐line, but also modifies the calcium and sodium currents of cardiomyocytes (Zheng et al., 2009). The suppression of ZASP/cypher protein induced by LDB3 pathogenic mutation was associated with DCM, left ventricular noncompaction (LVNC), arrhythmogenic cardiomyopathy (ACM) (Lopez‐Ayala et al., 2015), HCM (Fratev et al., 2014), heart failure (HF), and SCD (Vatta et al., 2003). The mutation of LDB3 p.M456R with MAF less than 0.001 located in exon 10 and predicted as “Damaging/Deleterious.” LDB3 p.M456R not only reduced its hydrophobicity of amino acid residues and their adjacent sequences, but also led to the transformation of 456^th^ amino acids from the non‐phosphorylation site to phosphorylation site, which therefore might change the function of ZASP protein.

The heart‐specific domain of ZASP/Cypher encoded by exon 10 of LDB3, preferentially expresses in the heart and binds to phosphoglucomutase 1 (PGM1) (Arimura et al., 2004). The pathogenic mutations in exon 10 of LDB3 may be associated with DCM via impaired the ZASP/Cypher binding to PGM1 (Arimura et al., 2009). PGM1 is a key enzyme in cellular glucose utilization and energy homeostasis, which support cardiac contractile activity and pathway of glucose metabolism (including glucose uptake and phosphorylation, glycolysis, and glucose oxidation) to produce an essential fuel under both normal and stress conditions in the heart (Depré et al., 1998; Sambandam et al., 2002). PGM1 translocating from cytoplasm and binding to ZASP/Cypher in the Z‐disks was enhanced in cardiomyocytes under cultured conditions of stress (Arimura et al., 2009). Therefore, we speculated that the mutation of LDB3 p.M456R in exon 10 would impair the cardiac glucose utilization and energy homeostasis, which therefore induced DCM, malignant ventricular tachycardia, and SCD as one of the key risk factors for this patient.

### 4.2 MYH6 and cardiomyopathy

Cardiac muscle myosin, along with actin, is one of the major components of sarcomere, the building block of cardiac contractile system. Myosin consists of two heavy chain subunits (alpha and beta), two light chain subunits and two regulatory subunits. MYH6 encodes the alpha myosin heavy chain subunit (α‐MHC) that encompasses ~26,000 bp and consists of 39 exons, 37 of which contain coding information. The α‐MHC consisting of head, neck, and tail domains plays a vital role in myofibril assembly and proper heart development. The tail domains of α‐MHC form a coiled coil that stabilizes the molecule so that the head domain is able to generate force through its interaction with actin (England & Loughna, 2013). MYH6 mutations cause HCM, DCM, and congenital heart disease with incomplete penetrance (Carniel et al., 2005; Ching et al., 2005; Granados‐Riveron et al., 2010; Gruner et al., 2011; Hershberger et al., 2010; Posch et al., 2011). The MYH6 p.R721T was associated with high risk of sick sinus syndrome (Holm et al., 2011). In three generations of Australian family, the MYH6 p.R654T resulted in early‐onset sinus node dysfunction, ventricular arrhythmias, and subsequent cardiac arrest (Lam et al., 2015). The compound heterozygosis for recessive MYH6 mutations (I704N/T1379M, D588A/E1207K) were demonstrated in patients with hypoplastic left heart and reduced right ventricular ejection fraction. The MYH6 p.I704N and p.D588A were located in the head domain and predicted to impair power stroke recovery and its interaction with actin, respectively. Additionally, the MYH6 p.T1379M and p.E1207K were located in the tail domain and predicted to affect the local structure of the coiled coil domain (Theis et al., 2015). The MYH6 p.A230P and p.A1366D disrupted myofibril formation, whereas the MYH6 p.H252Q enhanced myofibril assembly (Granados‐Riveron et al., 2010), which therefore induced atrial septal defects. Moreover, MYH6 knockdown quickly and efficiently induced DCM and HF (Carroll et al., 2016). In our study, the MYH6 p.S180Y were predicted as “Damaging/Deleterious” and not found in the population of 1000 Genomes Project. The MYH6 p.S180Y was located in the motor domain of myosin head, which associated with power stroke recovery and interaction with actin. The mutation reduced the hydrophobicity of α‐MHC, and meanwhile led to the disappearance of potential phosphorylation of the corresponding amino acid site, which may induce weaken force and interaction with actin, and subsequently participate into the pathogenicity of DCM.

### 4.3 SYNE1 and cardiomyopathy

SYNE1 encodes the nesprin, including 8,797 amino acids in human. Its N‐terminus is localized to cardiac sarcomeres, while the C‐terminus is localized to the nuclear envelope participating in a complex that links the nucleoskeleton to the cytoskeleton (LINC) (Zhang et al., 2002). The nesprin is involved in a force transmission between cytoskeleton and the nucleus and transduces the mechanical signal into transcriptional response through protein–protein interactions (Gu et al., 2017). SYNE1 mutations are associated with different cardiac phenotypes as DCM with conduction system defects (Puckelwartz et al., 2010), but also with slight left ventricular basal and septal hypertrophy with mild diastolic dysfunction (Zhang et al., 2007). According to previous reports, the SYNE1 p.P3992R (NM_033071: exon73:c.C11975G) was associated with emery‐dreifuss muscular dystrophy and HCM (Sandra et al., 2019). The SYNE1 mutations (p.R8272Q, p.S8381C, p.N8406K and p.R374H) increased levels of nesprin‐1α and Lamin A/C protein and disrupted LINC, which contributed to the pathogenesis of DCM (Puckelwartz et al., 2010; Zhou et al., 2017). The splice mutation (c.6403‐1) of SYNE1 inducing physical disruption of LINC complex interactions may be the plausible biological mechanism underlying the familial DCM (Haskell et al., 2017). The SYNE1 p.S4607F predicted as “Damaging/Deleterious” in our study was not found in the population of 1000 Genomes Project. It significantly increased the hydrophobicity of amino acid residues and their adjacent sequences, and meanwhile led to the disappearance of phosphorylation of the corresponding amino acid site. These changes of protein property and secondary structure of nesprin would lead to LINC disruption, which might participate into the pathogenesis of DCM and SCD in our study.

In the family of our study, the parents carrying with only SYNE1 p.S4607F or LDB3 p.M456R/MYH6 p.S180Y mutations presented with no phenotype of cardiomyopathy or arrhythmia. When fusing with these three mutations in the patient of II: 1, she occurred with young early‐onset and severe DCM, and subsequently died of VT/F even with ICD and anti‐arrhythmia therapy. The pairwise combinations of these three mutations have obvious and high joint pathogenicity, especially the combination of SYNE1/LDB3 and SYNE1/MYH6 mutations. It was worth noting that, the participation of SYNE1 p.S4607F may be the most important risk factor for DCM, VT/F and SCD, when carrying with LDB3 and MYH6 mutations. These three mutations induced early‐onset, exacerbate, progressive, and deteriorated phenotype of DCM involving in the bilateral ventricles, characterized by obviously atrophied, dissolved and disappeared of myocardial fibers; hyperplasia interstitial fibers, disrupted intercalated disk, vacuolar degeneration of mitochondria, accumulated and filled glycogen particles. The abnormal indirect protein–protein interaction induced by these three mutations through changing their hydrophobicity and phosphorylation of amino acids may contribute into the mechanism of young early‐onset DCM, VT/F and SCD.

### 4.4 Study limitations

Further animal or cell experiments are needed to confirm the mechanisms how these three mutations abnormally change the cardiac structure and function and result in the DCM and SCD.

## 5 CONCLUSIONS

We firstly reported that the multiple heterozygous mutations of SYNE1 p.S4607F, LDB3 p.M456R, and MYH6 p.S180Y with high joint pathogenicity may induce abnormal indirect protein–protein interactions through changing their hydrophobicity and phosphorylation of amino acids, which therefore led to young early‐onset and severe DCM, and subsequent SCD associated with VT/F.

## CONFLICT OF INTEREST

The authors declare that they have no competing interest.

## AUTHOR CONTRIBUTIONS

TZ and YBL: Pedigree analysis and writing; YTM: Bioinformatics analysis; ZQZ, JZX, and FW: Imaging analysis; ZY and XJG: Case collection and follow‐up; JNH: Sanger sequencing; YL and YBL: Quality control of clinical data and clinical design.

## ETHICAL APPROVAL

This study was approved by the Guangdong Medical Institutional Review Board and Medical Ethics Committees [No. GDREC2016001H (R1)]. With the consent of the ethics committee, we followed up the patients and their family members under the condition of informed consents and obtained blood samples for genetic analysis.

## Data Availability

The data used in this study are not publicly available, but it might be available from the corresponding author upon reasonable request and permission from relevant Chinese Authorities.
